# Do we parse the background into separate streams in the cocktail party?

**DOI:** 10.3389/fnhum.2022.952557

**Published:** 2022-10-28

**Authors:** Orsolya Szalárdy, Brigitta Tóth, Dávid Farkas, Gábor Orosz, István Winkler

**Affiliations:** ^1^Institute of Behavioural Sciences, Faculty of Medicine, Semmelweis University, Budapest, Hungary; ^2^Institute of Cognitive Neuroscience and Psychology, Research Centre for Natural Sciences, Budapest, Hungary; ^3^Unité de Recherche Pluridisciplinaire Sport Santé Société, Université d’Artois, Université de Lille, Université du Littoral Côte d’Opale, Liévin, France

**Keywords:** speech processing, background segregation, N2, P3, target detection

## Abstract

In the cocktail party situation, people with normal hearing usually follow a single speaker among multiple concurrent ones. However, there is no agreement in the literature as to whether the background is segregated into multiple streams/speakers. The current study varied the number of concurrent speech streams and investigated target detection and memory for the contents of a target stream as well as the processing of distractors. A male-voiced target stream was either presented alone (single-speech), together with one male-voiced distractor (one-distractor), or a male- and a female-voiced distractor (two-distractor). Behavioral measures of target detection and content tracking performance as well as target- and distractor detection related event-related brain potentials (ERPs) were assessed. We found that the N2 amplitude decreased whereas the P3 amplitude increased from the single-speech to the concurrent speech streams conditions. Importantly, the behavioral effect of distractors differed between the conditions with one vs. two distractor speech streams and the non-zero voltages in the N2 time window for distractor numerals and in the P3 time window for syntactic violations appearing in the non-target speech stream significantly differed between the one- and two-distractor conditions for the same (male) speaker. These results support the notion that the two background speech streams are segregated, as they show that distractors and syntactic violations appearing in the non-target streams are processed even when two speech non-target speech streams are delivered together with the target stream.

## Introduction

In everyday environments, we often attend to speech in the presence of multiple other speech streams (termed the “cocktail party” situation; Cherry, 1953). Typically, the listener’s goal is to follow the content of one speech stream while a speech from other talkers may distract him/her. People with normal hearing usually manage this situation (see, e.g., Bregman, 1990; Wood and Cowan, 1995). To this end, the auditory system must decompose the mixture of sounds into meaningful streams (auditory scene analysis; Bregman, 1990) and select the one with the behaviorally relevant information (selective attention; Best et al., 2008; Astheimer and Sanders, 2009). At the same time, processing of the irrelevant stream(s) should be suppressed to some degree in order to conserve capacities and prevent distraction (Ihlefeld and Shinn-Cunningham, 2008; Szalárdy et al., 2019, 2020b). Some studies showed that the auditory system can use a foreground-background solution with sounds in the acoustic background not being separated to further streams (e.g., Brochard et al., 1999; for a review, see Snyder and Alain, 2007). However, this might not be always the case, for instance when some of the background streams contain distinct auditory features (as suggested by Cusack et al., 2004; Winkler et al., 2012). In the current study, we tested whether two non-target speech streams are segregated in the presence of a third (target) speech stream. To this end, we assessed target detection and content tracking performance and the processing of distractors speech using behavioral measures and event-related brain potentials (ERPs).

Speech processing in the presence of other concurrent sound streams has been the target of several studies (for a recent review, see Bronkhorst, 2015). These studies mostly reported higher processing demand in the presence of concurrent speech compared to that with a single speech stream, as indicated by both behavioral and neural measures, which resulted from the masking effect of the distractor (see, e.g., Lambrecht et al., 2011). When speech was used for the distractor, stronger masking and reduced target detection performance were observed for the target speech stream compared to spectrally matched noise distractors (Kidd et al., 2005), as speech distractor masks the target not only energetically, but also informationally. Whereas the energetic masking component of a speech distractor influences the separation of the speech streams in a bottom-up manner (simply by the higher energy of the masker), informational masking can occur even when the streams are segregated, because of the similarity between the target and the masker, and uncertainty (Brungart et al., 2001; Arbogast et al., 2002). Thus, speech streams to be suppressed may lead to allocation errors through information masking. For example, when listeners were instructed to detect words in the target speech stream, there was a significant chance of reporting words from the distractor (masker) speech stream (Kidd et al., 2005; Wightman and Kistler, 2005). In our previous study (Szalárdy et al., 2019), listeners heard two concurrent speech streams, and they were instructed to detect numerals in the target stream. We found reduced detection sensitivity (d′), decreased hit, and increased false alarm rates with increased information masking. Furthermore, informational masking has been shown to influence the neural representation of the target speech (Szalárdy et al., 2019; Kawata et al., 2020).

The issue of auditory foreground-background decomposition has also been addressed by several experimental and theoretical papers (Siegenthaler and Barr, 1967; Teki et al., 2011; Tóth et al., 2016). Many of these papers suggest that when the auditory scene is segregated into streams, one of the streams can be consciously perceived, forming the auditory foreground while the rest of the auditory scene falls outside conscious perception, forming the background. This notion is, for example, supported by studies measuring the mismatch negativity (MMN, an event-related potential elicited by violations of acoustic regularities; for a recent review, see Fitzgerald and Todd, 2020), as some studies found MMN only to deviants violating regularities of the currently consciously experienced sound sequence (i.e., the foreground; Sussman et al., 1998; Winkler et al., 2006; Rahne et al., 2007).

Somewhat less is known about the extent to which the background stream is processed. Some studies showed that occasionally, sounds from the background may intrude into consciousness, for instance, some unexpected or personally relevant acoustic event (see, e.g., Micheyl et al., 2007), but not regularities, *per se* (Southwell et al., 2017). Furthermore, there is also evidence showing that violations of some regularities are detected also within the background stream (Szalárdy et al., 2013b) and, in general, stream segregation can occur outside the focus of attention (Bregman, 1990; Sussman et al., 2007). Thus, the question remains, whether sounds outside the focus of attention form an unsegregated background or the processing received by sounds outside the focus of attention includes stream segregation. Winkler et al. (2012) described the alternatives, arguing for a full segregation model (Mill et al., 2013). Here, we test this possibility for concurrent speech streams.

Event-related potentials (ERPs) were measured, because they allow one to study processes of target detection, attentional selection, working memory, and distraction. Target auditory events (including speech stimuli) typically elicit two successive ERP components, the N2 and the P3 (Näätänen et al., 1982; Ritter et al., 1983; Polich and Herbst, 2000; Polich, 2007). The N2b is a negative potential reaching maximal amplitude at around 200 ms from stimulus onset with a typical centro-parietal scalp distribution. In contrast to other subcomponents from the N2 family, N2b typically appears after a detected target event and has been associated with stimulus classification (Ritter et al., 1979; Näätänen, 1990). Studies have found that the amplitude of N2b is modulated by selective attention (Michie, 1984) and stream segregation (Szalárdy et al., 2013a). For generality, we refer to this component as N2. The N2 is often followed by the P3, which is a positive potential usually peaking between 300 and 400 ms from stimulus onset and with a parietally dominant scalp distribution (Polich, 2003; Conroy and Polich, 2007). P3 has been associated with context updating (Donchin and Coles, 1988), categorization, and later evaluation of the target stimulus (Nasman and Rosenfeld, 1990). The P3 has been shown to reflect the interaction between selective attentional processes and working memory (Polich and Herbst, 2000). This component has been selectively modulated by informational (and energetic) masking in our previous experiment (Szalárdy et al., 2019), resulting in reduced P3 when poorer allocation of attention could be assumed. Both components appear with larger amplitude with increased cognitive demand (Isreal et al., 1980; Polich, 2007). For non-target surprising events, another subcomponent from the P3 group is elicited, the P3a or novelty P3 (Polich, 2007). In a previous study, this component was elicited by target-like events appearing within a non-target speech stream delivered concurrently to the target speech stream (Szalárdy et al., 2020b). In the current study, N2 and P3 will be used to assess the effects of the manipulations on target detection and processing of distractors.

A continuous target speech stream was presented to the participants alone (single-speech condition) or in the presence of one or two continuous distractor speech stream(s): one condition with a male distractor voice (one-distractor condition) and one condition with a male and a female voice (two-distractor condition). The target stream was always delivered by a male speaker. Participants were instructed to detect numerals in the target stream by pressing a reaction key (target detection task). The distractor stream(s) also contained numerals (distractor events). Detection performance and ERPs were measured for targets together with false alarms caused and ERPs elicited by the distractor events, separately for each distractor stream (one-distractor male, two-distractor male, two-distractor female). Participants were also asked to follow the target speech and to answer questions based on information presented in it (recognition task). We hypothesized that performance (both target detection and recognition performance) will be lower in the conditions with distractor speech streams compared to the single-speech condition. Concurrently, based on previous studies showing that the amplitude of the N2/P3 amplitudes to target events increase with increasing task demand (Isreal et al., 1980; Polich, 2007; Szalárdy et al., 2020a), we hypothesized that the N2/P3 elicited by target numerals will be larger in the presence of distractor speech compared to the single-speech condition. By using two distractor speech streams delivered by speakers of different gender, we aimed to provide acoustically sufficiently distinct stimuli to promote segregation of the two background streams. The presence of two distractors may increase the energetic and/or information masking effect on the target stream, which should be measured in the target detection performance. If the two non-target streams are segregated, then one should expect performance and target-related ERP-amplitude decrease from the one- to the two-distractor condition due to the additional processes required for segregating the two non-target streams. Specifically, we expect that differences will be measured on the processing of the distractor male event, which is the same non-attended event in both the one- and two-distractor conditions. If this non-attended event is processed differently in the one- and two-distractor condition, that will suggest that non-attended streams are segregated from each other. In contrast, small or no performance and ERP differences between the one- and the two-distractor condition would suggest a predominantly foreground/background solution of the two-distractor condition by the auditory system.

## Materials and methods

### Participants

Native Hungarian speakers (*N* = 29; 11 males; age: *M* = 21.97 years, SD = 2.04; 26 right-handed) participated in the study for modest financial compensation. None of the participants had a history of psychiatric or neurological symptoms. All participants had pure-tone thresholds of <25 dB in the 250 Hz – 4 kHz range, with <10 dB difference between the two ears. Data from two participants were excluded from the final analysis due to the loss of the EEG triggers for sound onset. Data from four participants were excluded due to extensive artifacts and bad signal-to noise ratio. Thus, data from 23 participants were analyzed (8 male, 15 female, mean age: 21.91 years, SD: 2.23, 21 right-handed). Written informed consent was obtained from all participants. The study was approved by the United Hungarian Ethical Committee for Research in Psychology (EPKEB), and it was in full compliance with the World Medical Association Helsinki Declaration and all applicable national laws.

### Stimuli

Speech recordings of approximately 6 min duration were used as stimuli (soundtracks recorded at 48 kHz with 32-bit resolution, mean duration: 355.33 s, SD: 12.28, mean word number per stream: 636.41, SD: 84.87; mean number of phonemes per word: 6.48, SD: 0.29). Hungarian, emotionally neutral informative articles of news websites were delivered by professional actors (two male and one female speaker) recorded at 48 kHz with 32-bit resolution in the same room where the experiment was conducted.

All articles were reviewed by a dramaturge checking for correct syntax and natural flow of the text. The recorded speech was edited by a professional radio technician. The average RMS of the sound recordings was equalized to −32dBfs by VST-based attenuation after applying either −20 dB or −15 dB C3 compressors, depending on the dynamics of the actor reading. Audio recordings were presented by Matlab R2014a software (Mathworks Inc., Natick, MA, USA) with Psychtoolbox 3.0.10 on two Intel Core i5 PCs with ESI Julia 24-bit 192 kHz sound cards connected to Mackie MR5 mk3 Powered Studio Monitor loudspeakers. The speech streams were presented with a fixed loudness level of ∼70 dB SPL. Speech recordings were delivered from the same position as they were recorded in order to recreate the reverberation effects of the recording situation. Thus, room acoustics effects did not differ between recording and the experimental setup. Each loudspeaker corresponded to one speaker.

**FIGURE 1:**
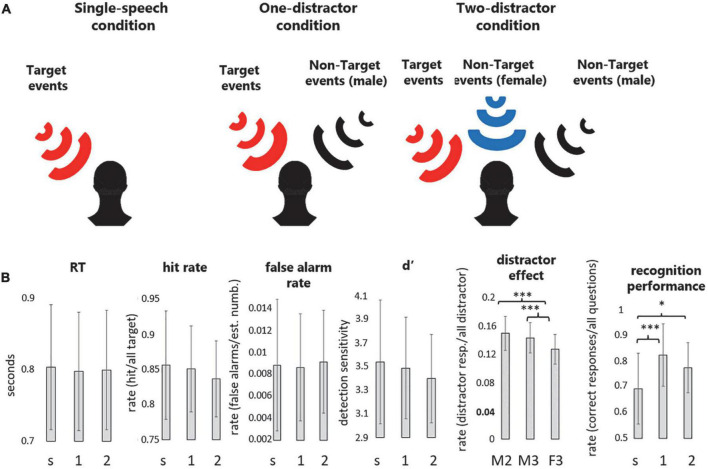
**(A)** Schematic illustration of the three experimental conditions. Participants were instructed to track the contents of the speech presented from the target location while also performing a numeral detection task on the same stream. In separate experimental conditions, participants were presented with (1) one speech stream (single-speech condition), (2) two speech streams (one-distractor condition), or (3) three speech streams (two-distractor condition). The target stream was always presented from the left of the listener (red loudspeaker). The black and blue wave symbols indicate the distractor male and distractor female streams, respectively. **(B)** Group average (*N* = 23) performance (mean and standard deviation) in the numeral detection task indexed by RT, hit rate, false alarm rate, d′, and the distractor effect, as well as recognition performance in the content-tracking task, separately for the three experimental conditions (s: single-speech condition, 1: one-distractor condition, 2: two-distractor condition). Note that because no distractor was present in the single-stream condition, the distractor effect was only calculated for the male speaker in the one-distractor (M2), the male speaker in the two-distractor (M3), and the female speaker in the two-distractor (F3) condition. *p < 0.05, ^***^p < 0.001, and ^+^0.05 < *p* < 0.1.

In three experimental conditions, one, two, or three speech streams were presented concurrently (see Figure 1A for a schematic illustration). Speech from the left loudspeaker (a male speaker’s speech) was designated as the target of the task (target stream). When delivered, the other stream(s) served as the distractor(s). Three conditions were created: the single-speech condition consisting of the single target male voice stream, the one-distractor condition consisting of the target and a distractor male voice stream, and the two-distractor condition consisting of the target male voice, a distractor male voice, and a distractor female voice stream. The spatial arrangement of the distractor streams was also constant throughout the experiment: the male actor’s speech was presented from the right, while the female actor’s speech from the central loudspeaker. The starting times of the audio playbacks for concurrent speech streams were synchronized by a microcontroller ensuring that all speech segments started within a 6 ms timeframe.

Each article contained 45–57 numerals (*M* = 50.7, SD = 2.7) of 2–4 syllable length. These served as targets in the target stream (targets) and distractors in the distractor stream(s). Only numerals indicating the quantity of some object within the context of the text were assigned as targets/distractors. For example, in Hungarian, the indefinite article (“egy”) is the same as the word for “one.” This word, when used as an article, did not constitute a target/distractor. There are also words, such as the Hungarian word for moonflower or daisy (“százszorszép” – literally translated as “hundred-times-beautiful”), which have a numeral as a component. These were not regarded as targets/distractors either. The temporal separation between successive target and distractor events was not controlled, because the articles serving as target and distractor streams were randomly paired, separately for each participant. In a representative example, the mean difference was calculated between target and distractor events: the mean difference was 2.348 s (SD: 1.722 s, min: 0.013 s, max: 7.535 s). Distractor articles (but targets not) also contained 19–26 syntactic violations (*M* = 20.5, SD = 1.4), which served for control purposes, as Szalárdy et al., 2018, 2020a found that when participants follow one of two concurrent speech streams, syntactic violations within the unattended stream do not elicit the syntax-violation related ERP components. Therefore, syntactic violations could be used to indicate whether the non-target stream(s) were attended or not.

Altogether 12 stimulus blocks were created from the 24 different articles. Each condition received four stimulus blocks. No article was presented twice to the same participant.

### Experimental procedure

The study was conducted in an acoustically attenuated, electrically shielded, dimly lit room at the Research Centre for Natural Sciences, Budapest, Hungary. Three loudspeakers were placed at an equal 200 cm distance from the participant, positioned symmetrically at −30° (left) 0° (middle), and 30° (right) from the midline. Additionally, a 23″ monitor was placed at 195 cm in front of the participant, showing an unchanging fixation cross (“+”) during the stimulus blocks. Participants were instructed to avoid eye blinks and other muscle movements and to watch the fixation cross while listening to the speech segments. EEG was recorded during the experimental blocks.

Participants performed two tasks on the target speech segments (Figure 1): the “numeral detection” and the “content tracking” task. In the numeral detection task, participants were instructed to press a hand-held response key with their right thumb as soon as they detected the presence of a numeral word (target events, see above). For the content tracking task, they were informed that at the end of each stimulus block, they will have to answer five questions regarding the contents of the target speech segment. Each question corresponded to a piece of information that appeared within the target speech segment. The experimenter read the question and the four possible answers. The listener was then asked to verbally indicate his/her choice for the correct answer (multiple-choice test). The experimenter noted the participant’s choice and followed up with a request for the participant to assess his/her confidence for the choice from four alternatives: “I don’t remember I was just guessing” (coded as 1), “I am not sure, but the option I chose sounded familiar: I think I heard it during the last block” (2), “I am sure; I remember having heard it during the last block” (3), “I know the answer from some other source” (0). The confidence rating was recorded by the experimenter. The two concurrent tasks served complementary purposes in directing the listener’s attention: Whereas the tracking task required listeners to integrate information over longer periods of time and to fully process the target speech segments, the detection task ensured that attention was continuously focused on the target speech segment.

The stimulus blocks were presented in pseudorandomized order: in the first half of the experimental session (blocks 1–6), each condition was presented two times in random order with the restriction that no condition was immediately repeated; in the second half of the session (blocks 7–12), conditions were presented in reversed order with respect to the first half. Participants were allowed to take a break during the experiment after each stimulus block, and there was a longer mandatory break after the sixth stimulus block. Altogether, the experiment lasted ca. 4 h.

### Behavioral data recording and analysis

Detection task performance: Button presses for correct responses (hits) were initially collected from a 0–5000 ms interval from the onset of the target event. Responses were then rejected if they were longer than 95% (>1493 ms) or shorter than 5% (<435 ms) of all of the initially collected potential target responses (collapsed across all conditions and participants). From the accepted responses, log-normalized reaction times (RT) and hit rates (HR) were calculated for each participant and condition (pooling data from the four stimulus blocks of the same condition). False alarm rates (FA) were calculated by dividing the number of non-target responses (any response outside the periods calculated for targets) by the estimated number of non-target words in the target-stream (calculated from the mean word length for all speech material used in the experiment). Detection sensitivity values (d′; Green and Swets, 1966) were calculated from HR and FA. The distractor effect was assessed for distractor numerals: the number of distractors with a button press response (from the same time-interval as was found for the corresponding targets) was divided by the number of all distractors, separately for each condition and distractor source (one-distractor male, two-distractor male, two-distractor female).

Recognition performance in the content-tracking task was calculated as the percentage of correct responses, separately for each participant and condition (pooling data from the four stimulus blocks of the same condition). The sensitivity of the measurement was increased by eliminating items (questions), the response to which was above 95% or below 30% correct overall (collapsed across participants and conditions). Responses with a confidence rating of “I know the answer from some other source” were also dropped from the analysis. Confidence ratings were compared between the three conditions (single-speech, one-distractor, two-distractor) by the Kruskal–Wallis *H* test, followed by Bonferroni-corrected pairwise *post hoc* contrasts.

Statistical analysis consisted of repeated-measures analyses of variance (ANOVA) with the factor of CONDITION (single-speech vs. one-distractor vs. two-distractor), separately for RT, d′, hit rate, false alarm rate, and recognition performance. Statistical analysis of distractor effect (assessed for distractor numerals, see the section “Materials and methods”) was performed by another repeated-measures ANOVA, with the factors DISTRACTOR (one-distractor male, two-distractor male, two-distractor female). The alpha level was set at 0.05. Greenhouse–Geisser correction of sphericity violations was employed where applicable and the ε correction factor is reported. All significant results are reported together with the η^2^ effect size. All statistical analyses (behavioral and ERP) were conducted by the STATISTICA 13.1 and JASP 0.15.0.0. software.

### EEG data recording and analysis

EEG recording and analysis were identical to Szalárdy et al. (2018, 2020a). Continuous EEG was recorded (1 kHz sampling rate and 100 Hz online low-pass filter) from a few seconds before the beginning to a few seconds after the end of the stimulus blocks using a BrainAmp DC 64-channel EEG system with actiCAP active electrodes (Brain Products GmbH, Gilching, Germany). EEG signals were synchronized with the speech segments by matching an event trigger marked on the EEG record to the concurrent presentation of a beep sound in the audio stream (1 s before the speech segment commenced) with <1 ms accuracy. Electrodes were attached according to the extended International 10/20 system with an additional electrode placed on the tip of the nose. For identifying eye-movement artifacts, two electrodes were placed lateral to the outer canthi of the two eyes. Electrode impedances were kept below 15 kΩ. The FCz electrode served as the online reference.

Continuous EEG data were filtered with a 0.5–80.0 Hz Kaiser bandpass-filter and a 47.0–53.0 Hz Kaiser bandstop filter (the latter for removing electric noise; Kaiser β = 5.65, filter length 18112 points) using the EEGlab 14.1.2.b toolbox (Delorme et al., 2007). EEG data processing was performed by Matlab R2018b (Mathworks Inc., Natick, MA, USA). Electrodes showing long continuous or a large number of transient artifacts were substituted using the spline interpolation algorithm implemented in EEGlab. The maximum number of interpolated channels was two per participant. The Infomax algorithm of Independent Component Analysis (ICA) implemented in EEGlab was employed for eye-movement artifact removal. Maximum 6 ICA components (approximately 10% of all components) constituting blink artifacts and horizontal eye-movements were removed via visual inspection of the topographical distribution and frequency contents of the components. Data were then offline re-referenced to the electrode attached to the tip of the nose. Epochs were extracted from continuous EEG records for a window of −200 – 2400 ms with respect to the onset of numerals. Numeral onsets were manually marked by a linguistic expert after automatic segmentation of the speech by Praat (version 6.0.20). Baseline correction was applied using the 200-ms pre-event interval. Artifact rejection with a threshold of ±100 μV voltage change was applied to the whole epoch, separately for each electrode. Artifact-free epochs were then averaged separately for each participant and condition. For target events, only hits, for distractors, only correct rejections were analyzed.

Amplitudes were measured from frontal (F3, Fz, F4), cental (C3, Cz, C4), and parietal (P3, Pz, P4) electrodes for statistical analysis, allowing also to compare response amplitudes across the left (F3, C3, P3), midline (Fz, Cz, Pz), and right (F4, C4, P4) areas. Time windows for measuring the ERP amplitudes were selected between 150 and 280 ms for N2 and between 620 and 770 ms for P3 relative to stimulus onset, both for target and non-target numerals. The average number of artifact-free target numerals were 150.17 (SD: 24.82) for the single-speech, 156.13 (SD: 24.77) for the one-distractor, and 160.39 (SD: 23.30) for the two-distractor condition. For distractor numerals and syntactic violations the average number of artifact-free trials were 179.26 (numeral, SD: 24.77) and 74.13 (syntactic violation, SD: 9.09) for the one-distractor condition, 171.17 (numeral, SD: 21.92) and 72.52 (syntactic violation, SD: 8.81) for the two-distractor male, and 184.26 (numeral, SD: 21.26) and 72.30 (syntactic violation, SD: 8.88) for the two-distractor female speaker.

Target ERP amplitudes were statistically analyzed using repeated-measures ANOVAs with the factors of CONDITION (single-speech, one-distractor, two-distractor) × ANTERIOR-POSTERIOR (frontal, central, parietal) × LATERALITY (left, middle, right), separately for the N2 and P3 components. Distractor ERPs and ERPs for syntactic violations were analyzed similarly, using repeated-measures ANOVAs with the factors of DISTRACTOR (one–distractor male, two-distractor male, two-distractor female) × ANTERIOR-POSTERIOR (frontal, central, parietal) × LATERALITY (left, middle, right), separately for the N2 and P3 components. *Post-hoc* tests were conducted for all main effects and interactions that included the CONDITION (for targets) or the DISTRACTOR (for distractors) factor by Tukey’s HSD. Greenhouse–Geisser correction of sphericity violations was employed where applicable and the ε correction factor is reported together with the η^2^ effect size. Only significant effects including the CONDITION/DISTRACTOR factor are reported in the main text (see the Supplementary Tables 1–6 of all ANOVA effects). In addition, for assessing whether numeral distractors and syntactic violations were processed in the non-target streams, the corresponding ERP amplitudes were compared to zero by one-sample Student’s *t*-tests.

## Results

### Behavioral measures

The descriptive statistics of the behavioral performance are shown in Figure 1B. A significant effect of condition was found for recognition performance [*F*(2,44) = 12.246, $\eta_{\text{p}}^{2}$ = 0.358, ε = 0.798, *p* < 0.001]. This was due to the significantly lower memory performance in the single-speech condition compared to both the one- (*p* < 0.001) and the two-distractor (*p* = 0.011) conditions, whereas the latter two did not significantly differ from each other (*p* = 0.161). No significant effects were found for the reaction times (*p* = 0.633), hit (*p* = 0.123), and false alarm rates (*p* = 0.676), while a marginally significant effect was obtained for detection sensitivity (*p* = 0.074).

A significant effect of DISTRACTOR was found on the distraction effect [*F*(2,44) = 19.200, $\eta_{\text{p}}^{2}$ = 0.466, ε = 0.862, *p* < 0.001]. The effect was caused by the significantly lower distractor effect of the numerals spoken by the two-distractor female speaker compared to the one- and two-distractor male speaker (*p* < 0.001, both). The latter two were not significantly different from each other (*p* = 0.225).

The confidence judgment was significantly different between the three conditions (Chi square = 34.189, *p* < 0.001, df = 2). *Post hoc* significance values were adjusted by the Bonferroni correction for multiple tests, showing that significantly larger confidence judgment occurred in the one-distractor condition compared to the single-speech and two-distractor conditions (*p* < 0.001, both) whereas these were not different from each other (*p* = 1.00).

### Event-related potential measures

**FIGURE 2:**
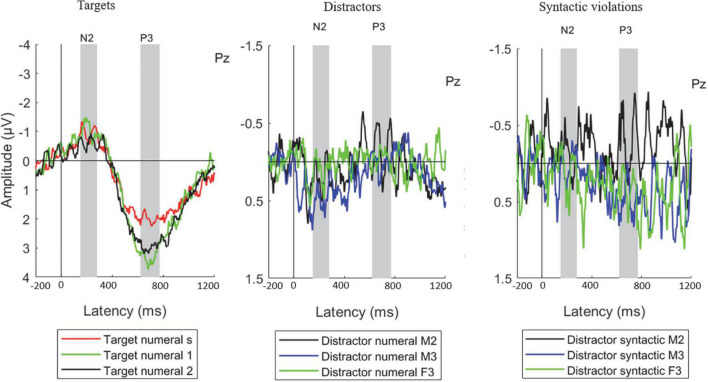
Group average (*N* = 23) ERP responses measured from the parietal (Pz) electrode position, separately for the target numerals **(left)**, distractor numerals **(middle)**, and distractor syntactic violations **(right)**. The measurement time windows for N2 and P3 are marked by gray vertical bands. Legend abbreviations for the target numerals: s – single-speech condition, 1 – one-distractor condition, 2 – two-distractor condition. Legend abbreviations for the distractor numerals and syntactic violations: M2 – male speaker in the one-distractor condition, M3 – male speaker in the two-distractor condition, F3 – female speaker in the two-distractor condition. Note the different scales for target and distractor responses.

**FIGURE 3:**
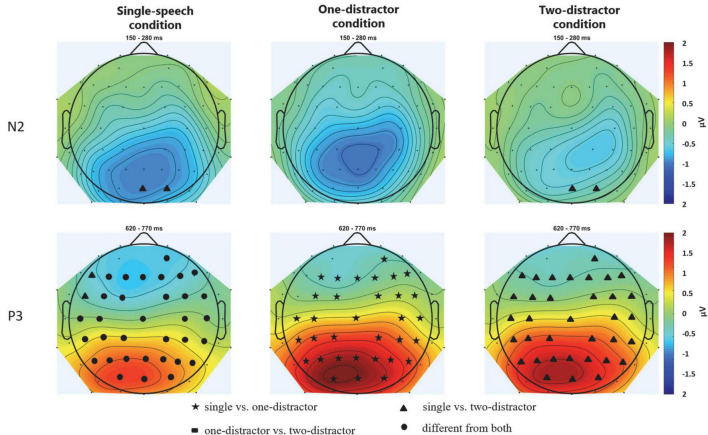
Scalp topography of the N2 **(upper row)** and P3 **(bottom row)** component for target numerals in the three experimental conditions: single-speech **(left)**, one-distractor **(middle)**, and two-distractor **(right)**. The scalp distributions were calculated from the average voltages measured from the time windows shown in Figure 2. Maps were spline interpolated with a smoothing factor of 10^–7^. Electrode locations marked by filled circles, stars, triangles, and rectangles represent the scalp locations where the signal in the given latency range significantly differed from one (filled stars, rectangles, or triangles) or both (circles) other conditions. Tests were computed by pair-wise *post hoc* comparisons of the corresponding ANOVA with the factors of CONDITION and ELECTRODE LOCATION (including all electrodes; thus replacing the factors ANTERIOR-POSTERIOR and LATERALITY).

Event-related potentials measured at the Pz electrode are shown on Figure 2. The scalp distributions of the target N2 and P3 show maximal amplitude for both components over parietal scalp locations (Figure 3), as was also seen in our previous studies (Szalárdy et al., 2018, 2019, 2020a,b).

#### Event-related potentials to targets

For target events, significant interaction was found for the N2 amplitude between CONDITION and LATERALITY [*F*(2,44) = 2.862; ε = 0.799; *p* = 0.0398; $\eta_{\text{p}}^{2}$ = 0.115]. *Post-hoc* tests showed that the N2 amplitude significantly differed between all three conditions (single-speech, one-distractor, two-distractor) on the left side (*p* = 0.0351, at least): the largest N2 amplitude was observed for one-distractor which decreased for single-speech with the lowest amplitude for two-distractor (best seen on Figure 3). In contrast, the N2 amplitudes for single-speech and one-distractor conditions were not significantly different from each other at the midline (*p* = 0.88) or on the right side (*p* = 1.00) with those for two-distractor condition significantly differing from both at the midline (*p* < 0.001, both). In all of these cases, the amplitude for the target N2 was lower for two-distractor condition than for single-speech and one-distractor condition. A significant main effect of CONDITION was found for the P3 component [*F*(2,44) = 18.580; ε = 0.987 *p* < 0.001; $\eta_{\text{p}}^{2}$ = 0.458] with interactions between CONDITION and LATERALITY [*F*(4,88) = 3.973; ε = 0.827; *p* = 0.009; $\eta_{\text{p}}^{2}$ = 0.153], and CONDITION, LATERALITY, and ANTERIOR-POSTERIOR [*F*(8,176) = 2.575; ε = 0.638; *p* = 0.029; $\eta_{\text{p}}^{2}$ = 0.105]. As P3 is maximal over parietal sites, for *post hoc* analysis, a separate ANOVA was conducted on the parietal line, alone, with the factors of CONDITION and LATERALTY. In this *post hoc* analysis, main effects of CONDITION [*F*(2,44) = 15.956; ε = 0.859; *p* < 0.001; $\eta_{\text{p}}^{2}$ = 0.420] and LATERALITY [*F*(2,44) = 13.921; ε = 0.763; *p* < 0.001; $\eta_{\text{p}}^{2}$ = 0.388] were found with no interaction between them (*p* = 0.292). The *post-hoc* test of the CONDITION main effect showed significantly lower amplitudes for single-speech condition compared to one-distractor and two-distractor (*p* < 0.001, both), while the latter two were not significantly different from each other (*p* = 0.204).

Based on the similar pattern between the recognition performance data and P3 amplitude in the three conditions, Pearson correlation was calculated between them, using the P3 measured at the Pz electrode. No significant correlation was found between the P3 amplitude and recognition performance in the single stream (*r* = −0.009; *p* = 0.966), one-distractor (*r* = −0.112; *p* = 0.621), and two-distractor conditions (*r* = −0.376; *p* = 0.077).

#### Event-related potentials to distractors and syntactic violations

Event-related potential amplitudes significantly different from zero were found in the N2 time window at the C3 (*p* = 0.030), Cz (*p* = 0.021), and C4 (*p* = 0.018) electrodes for distractor numerals appearing in the male-spoken stream of the two-distractor condition. No other distractor numeral or syntactic violation ERP amplitudes differed significantly from zero in the N2 latency range (*p* > 0.072, at least). In the P3 time window, ERP amplitudes significantly differing from zero were found for distractor numerals in the one-distractor condition (electrodes: F3, *p* = 0.048; Fz, *p* = 0.031; F4, *p* = 0.047), and for syntactic violations appearing in the male-spoken non-target speech stream in the two-distractor condition (electrodes: C3, *p* = 0.008; Cz, *p* = 0.046; Pz, *p* = 0.047; P4, *p* = 0.035). No other distractor numeral or syntactic violation ERP amplitudes differed significantly from zero in the P3 latency range (*p* > 0.056, at least).

**FIGURE 4:**
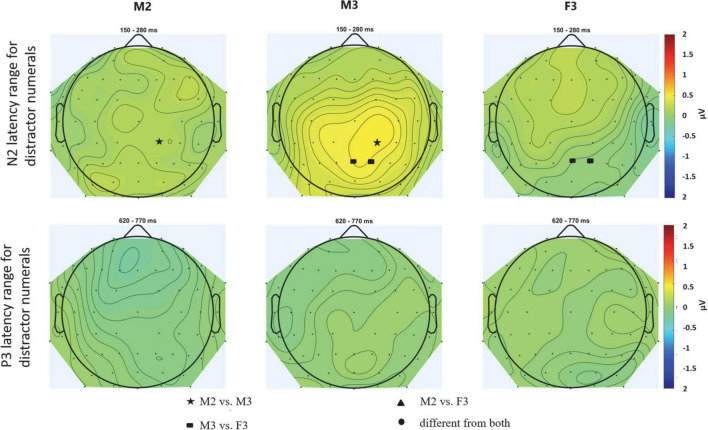
Scalp topography of the N2 **(upper row)** and P3 **(bottom row)** latency range for distractor numerals for the three distractor streams: M2 – male speaker in the one-distractor condition, M3 – male speaker in the one-distractor condition, F3 – female speaker in the two-distractor condition. The scalp distributions were calculated from the average voltages measured from the time windows shown in Figure 2. Maps were spline interpolated with a smoothing factor of 10^–7^. Electrode locations marked by filled circles, stars, triangles, and rectangles represent the scalp locations where the signal in the given latency range significantly differed from one (filled stars, rectangles, or triangles) or both (circles) other conditions. Tests were computed by pair-wise *post hoc* comparisons of the corresponding ANOVA with the factors of CONDITION and ELECTRODE LOCATION (including all electrodes; thus replacing the factors ANTERIOR-POSTERIOR and LATERALITY).

The positive deflection measured for the distractors in the N2 time window (Figure 2, middle; Figure 4 for scalp distributions), a significant DISTRACTOR × ANTERIOR-POSTERIOR interaction was found [*F*(4,88) = 3.429; ε = 0.547; *p* = 0.037; $\eta_{\text{p}}^{2}$ = 0.135]. *Post-hoc* tests revealed that this interaction was due to the central ERP amplitude in the N2 time window for two-distractor male being more positive than that for one-distractor male centrally (*p* = 0.033), and also than one-distractor male and two-distractor female parietally (*p* = 0.009, both). In contrast, no significant difference was found for the amplitudes from the N2 window between the one-distractor male and two-distractor female either over central (*p* = 0.491) or parietal electrode locations (*p* = 1.000). No other significant difference was found either in the N2 or the P3 time window.

**FIGURE 5:**
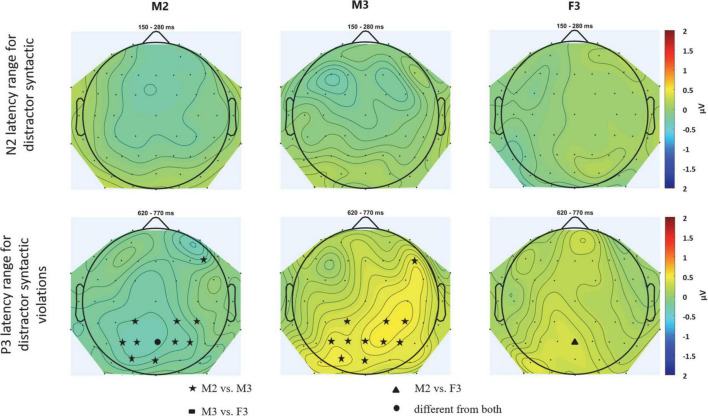
Scalp topography of the N2 **(upper row)** and P3 **(bottom row)** latency range for distractor syntactic violations for the three distractor streams: M2 – male speaker in the one-distractor condition, M3 – male speaker in the one-distractor condition, F3 – female speaker in the two-distractor condition. The scalp distributions were calculated from the average voltages measured from the time windows shown in Figure 2. Maps were spline interpolated with a smoothing factor of 10^–7^. Electrode locations marked by filled circles, stars, triangles, and rectangles represent the scalp locations where the signal in the given latency range significantly differed from one (filled stars, rectangles, or triangles) or both (circles) other conditions. Tests were computed by pair-wise *post hoc* comparisons of the corresponding ANOVA with the factors of CONDITION and ELECTRODE LOCATION (including all electrodes; thus replacing the factors ANTERIOR-POSTERIOR and LATERALITY).

Finally, there was no significant main effect or interaction for syntactic violations in the N2 time window (*p* > 0.172, at least; Figure 2, right panel; see also Figure 5 for scalp distribution). However in the P3 time window, a significant main effect of DISTRACTOR was found [*F*(2,44) = 3.774; ε = 0.908; *p* = 0.031; $\eta_{\text{p}}^{2}$ = 0.146], whereas no other main effect or interaction was significant (*p* > 0.067). *Post-hoc* test revealed that the main effect resulted from the more positive deflection for the two-distractor male than for the one-distractor male (*p* = 0.030) syntactic violations, whereas none of them was different from the two-distractor female (*p* > 0.133).

Participants of different gender in this study could be affected differently by the gender of the target and distractor voices. Therefore, the main statistical analyses were repeated with the participant’s gender as a grouping variable (see Supplementary Results).

## Discussion

We investigated whether multiple distractor speech streams are segregated from each other and their effects on the lexical/semantical processing of the target speech stream. The current data corroborated previous findings (Bronkhorst, 2015) in that the P3 amplitude increased from the single speech to the concurrent speech streams conditions. Importantly, the behavioral distractor effect differed between the conditions with one vs. two distractors (distraction by the female speaker was lower than that of the male speaker in either condition) and the target N2 elicited in the presence of two distractors was significantly smaller than that elicited in the presence of one distractor. Further, both the positive deflection in the N2 time window to distractors and the response to syntactic violations significantly differed between the one- and two-distractor conditions for the same male speaker (see Figures 2, 4, 5). These results show that speech processing was different in the presence of one vs. two distractors, and thus, in terms of the alternatives described in the introduction, the current data suggest that the two background speech streams were segregated from each other.

According to our hypothesis, if the two background streams were segregated, the distractor events should be processed differently in the one- and two-distractor condition (cf., Cusack et al., 2004; Winkler et al., 2012). Thus the strongest evidence supporting this hypothesis come from the non-zero voltage for distractor numerals and syntactic violations in the two-distractor condition and the significantly different amplitudes observed in the N2 (for distractor numerals) and P3 (for syntactic violations) time windows for the same distractor male speaker in the presence vs. the absence of the stream delivered by the female speaker. The former reflects that numerals and syntactic violations appearing in non-target streams were processed even when two such streams were delivered, suggesting that the two non-target streams were segregated from each other. The latter suggests that distractors are processed differently alone than in the presence of another distractor stream. If we assume that the responses in the N2 latency range reflect target identification processes, then the differential response to the same distractor between the one- and the two-distractor condition reflects that target identification (rejection of the distractor) within the male distractor stream proceeded under a higher processing load due to the presence of the second distractor stream (i.e., the target stream was present in both conditions). The presence of another distractor results in higher information density and thus the allocation of reduced capacities to each stream, which in turn modulates both the target N2 (as discussed before) and the processes in the N2 range of the distractors (see Isreal et al., 1980; Conroy and Polich, 2007; Dowling et al., 2008; Szalárdy et al., 2019) as well as the processes in the P3 range of the responses to syntactic violations for the distractor streams. Crucially, identifying and rejecting target candidates in a distractor stream requires the stream to be segregated from both the target and the other distractor stream. Therefore, the results support the notion of segregating the background in the current situation. This conclusion is compatible with models suggesting full object-based description of the environment (for a general model of learning, see, Fiser, 2009; in the auditory modality, see e.g., Winkler et al., 2012; Mill et al., 2013).

Based on the behavioral results, the smaller distracting effect of the female voice alone may be explained in the context of both alternatives. It is compatible with the notion of segregating the two background streams with the additional assumption that distractors in the female voice were less likely to be confused with the target spoken in a male voice than those of another male voice. However, one could also assume that within the undifferentiated background, the female voice was a less effective masker for the target stream. Previous studies showed that target detection is reduced when a speech segment is masked by high-level noise (energetic masking), whereas allocation problems can be found when a speech stream is masked by another speech (informational masking; Darwin, 2008; Ihlefeld and Shinn-Cunningham, 2008). The current results showed no significant hit rate or false alarm difference between the one- and two-distractor conditions. Because the male distractor was common to both conditions, the lack of significant change in task performance is compatible with the less effective masker explanation. Note that, because the location of the two distractor stream sources was not varied, distractor gender and source location are confounded. However, the female voiced stream was presented from the centrally located loudspeaker, which was thus spatially closer to the target stream than the source of the male voiced distractor stream. This suggests that spatial separation has a smaller effect on distraction by a concurrent stream than voice similarity.

The undifferentiated background hypothesis is, however, contrasted by both the target N2 and the distractor and syntactic violation ERP amplitudes measured from the N2 and P3 windows, respectively. The N2 is a target-related response that has been associated with stimulus classification (Ritter et al., 1979; Näätänen, 1990) and its amplitude is modulated by selective attention (Michie, 1984). In contrast to our hypotheses, we found that the amplitude of the N2 did not linearly increase with task demand, but increased for the one-distractor conditions and decreased for the two-distractor condition. The decrease of the target N2 amplitude suggests that the identification of targets (the assumed role of the processes reflected by N2 –Ritter et al., 1979; Näätänen, 1990) differed between the one-distractor and two-distractor conditions. The two conditions were different in the background streams only, and the target properties were identical. The distractor stream thus served as a masker, which could have energetically and informationally masked the target stream. If masking (whether energetic or information) was the only way target identification was affected, then the N2 amplitude should have corresponded to the behavioral effects, mirroring the lack of difference found for P3. Furthermore, in a previous study, the N2 was not sensitive to the effect of informational and energetic masking, but rather, the amplitude was modulated by attention (Szalárdy et al., 2019). The significant N2 difference observed may be explained by attentional selection: assuming that in a multi-stream situation, target detection must also include validating targets by taking into account the stream the candidate belongs to. Alternatively, results showing that enhancing cortical tracking of ignored speech by transcranial alternating current stimulation reduces comprehension of the target stream (Keshavarzi et al., 2021) suggests that in the current study, cortical tracking of background speech for one vs. two speech streams differentially affected the segregation of the target stream. This alternative receives support from the similar pattern of N2 amplitude and confidence ratings (indexing comprehension).

However, the current data do not prove that the background is always parsed into its constituent streams. There are studies showing that a background consisting of potentially separable streams remained undistinguished (e.g., Brochard et al., 1999; Sussman et al., 2005). The crucial difference between these and the current study is the type of sounds presented in the different streams. While the studies, which found no segregation of streams in the background delivered simple sounds (mainly pure tones) differing from each other in one feature, here we presented natural speech, and specifically, the two non-target streams differed in the speaker’s gender, making them highly distinctive. In a recent study, attended and ignored speech streams were both represented in the auditory cortex (mostly in primary areas), suggesting the global representation of the full auditory scene with all auditory streams (Puvvada and Simon, 2017). Other studies also confirmed the recognition of some words from a background speech stream, even if the background consisted of multiple voices (Dekerle et al., 2014). Furthermore, signs of spectro-temporal and linguistic processing of task-irrelevant speech streams were found in the auditory cortex, left inferior cortex, and posterior parietal cortex (Brodbeck et al., 2020; Har-shai Yahav and Zion Golumbic, 2021). The prerequisite of background stream segregation might be highly distinctive features, which results in categorical differences, such as different gender of speakers; but this background stream segregation might be unique to speech perception. Cusack et al. (2004) have already speculated that distinctive acoustic features could allow streams to be segregated outside the focus of attention, and several studies have shown stream segregation when none of the streams was specifically attended (e.g., Sussman et al., 2005; Sussman, 2007). It is, therefore, possible that segregation of the background depends on both the perceptual difficulty of the separation (Cusack et al., 2004; Keshavarzi et al., 2021) and the available capacity (Sussman et al., 2005). A control condition with two male distractors or two-non-speech distractor streams could provide further evidence regarding the segregation of background speech streams, and whether separation requires distinct acoustic features such as different gender of the speakers. Thus, future studies are needed to shed light on the prerequisite of background stream segregation.

Somewhat surprisingly, recognition performance was significantly lower in the single-speech condition compared to the conditions with distractors. This pattern of results was accompanied by a corresponding P3 amplitude effect, and the N2 was also lower for this condition suggesting poorer allocation of attention. Furthermore, the confidence judgment was also lower in the single-speech condition compared to the one-distractor condition, but not in the two-distractor condition. Similar correspondence was found between recognition performance and the P3 amplitude in our previous experiment based on similar methods but presenting only a single distractor (Szalárdy et al., 2019). This is not a trivial finding, as P3 was elicited in a task (numeral detection) concurrent to the memory task (which was only tested after the stimulus blocks). Studies testing working memory also found that better performance was associated with higher P3 amplitude, especially with higher motivational salience (e.g., reward, punishment; Baskin-Sommers et al., 2014) and for young healthy adults (Saliasi et al., 2013). Thus, it is possible that performing the tasks under more difficult circumstances [in the presence of distractor stream(s)] resulted in better engagement with the task, which boosted performance in content tracking. Alternatively, performing the target detection task at a high level in the presence of distractor streams forced participants to utilize higher-level speech cues (syntactic and semantic) in order to determine whether a given numeral (candidate target) indeed needed a response. Investing more effort in fully processing the target speech stream could have resulted in better memory for the contents of the speech stream, and thus higher recognition performance. Although the current results do not allow us to separate the alternative explanations, they corroborate the previously observed correspondence between recognition memory performance and the P3 amplitude.

## Data availability statement

The original contributions presented in this study are included in the article/Supplementary material, further inquiries can be directed to the corresponding author.

## Ethics statement

The studies involving human participants were reviewed and approved by United Hungarian Ethical Committee for Research in Psychology (EPKEB). The patients/participants provided their written informed consent to participate in this study.

## Author contributions

IW, GO, and BT contributed to the conception and design of the study. DF organized the database. OS performed the statistical analysis and wrote the first draft of the manuscript. BT and IW wrote sections of the manuscript. All authors contributed to the manuscript revision, read, and approved the submitted version.
