# Supplementary material for: Do we parse the background into separate streams in the cocktail party?
Source: Front Hum Neurosci. 2022 Oct 28;16:952557. doi: 10.3389/fnhum.2022.952557 (PMC9649784; doi:10.3389/fnhum.2022.952557)
Supplement: Supplementary file 1 [file Data_Sheet_1.docx]

**Supplementary Table 1. ANOVA effects for target N2**

| Effect | Repeated Measures Analysis of Variance of target N2 | | | | | | | |
| --- | --- | --- | --- | --- | --- | --- | --- | --- |
|  | SS | Degr. of (Freedom) | MS | F | p | Partial eta-squared | Non-centrality | Observed power (alpha=0,05) |
| Intercept | 250.898 | 1 | 250.898 | 24.391 | 0.000 | 0.526 | 24.391 | 0.997 |
| Error | 226.300 | 22 | 10.286 |  |  |  |  |  |
| F-C-P | 40.463 | 2 | 20.231 | 14.231 | 0.000 | 0.393 | 28.461 | 0.998 |
| Error | 62.553 | 44 | 1.422 |  |  |  |  |  |
| L-M-R | 1.559 | 2 | 0.779 | 2.881 | 0.067 | 0.116 | 5.762 | 0.535 |
| Error | 11.903 | 44 | 0.271 |  |  |  |  |  |
| S-1-2 | 8.823 | 2 | 4.411 | 1.193 | 0.313 | 0.051 | 2.387 | 0.248 |
| Error | 162.664 | 44 | 3.697 |  |  |  |  |  |
| F-C-P*L-M-R | 2.218 | 4 | 0.554 | 5.870 | 0.000 | 0.211 | 23.480 | 0.979 |
| Error | 8.311 | 88 | 0.094 |  |  |  |  |  |
| F-C-P*S-1-2 | 1.324 | 4 | 0.331 | 1.087 | 0.368 | 0.047 | 4.348 | 0.329 |
| Error | 26.794 | 88 | 0.304 |  |  |  |  |  |
| L-M-R*S-1-2 | 0.974 | 4 | 0.244 | 2.862 | 0.028 | 0.115 | 11.447 | 0.756 |
| Error | 7.489 | 88 | 0.085 |  |  |  |  |  |
| F-C-P*L-M-R*S-1-2 | 0.208 | 8 | 0.026 | 0.718 | 0.676 | 0.032 | 5.743 | 0.327 |
| Error | 6.360 | 176 | 0.036 |  |  |  |  |  |

**Supplementary Table 2. ANOVA effects for distractor N2 time-window**

| Effect | Repeated Measures Analysis of Variance of distractor in the N2 time-window | | | | | | | |
| --- | --- | --- | --- | --- | --- | --- | --- | --- |
|  | SS | Degr. of (Freedom) | MS | F | p | Partial eta-squared | Non-centrality | Observed power (alpha=0,05) |
| Intercept | 8.693 | 1 | 8.693 | 2.230 | 0.150 | 0.092 | 2.230 | 0.298 |
| Error | 85.767 | 22 | 3.898 |  |  |  |  |  |
| F-C-P | 0.519 | 2 | 0.259 | 0.556 | 0.577 | 0.025 | 1.113 | 0.136 |
| Error | 20.518 | 44 | 0.466 |  |  |  |  |  |
| L-M-R | 0.167 | 2 | 0.083 | 0.602 | 0.552 | 0.027 | 1.205 | 0.144 |
| Error | 6.092 | 44 | 0.138 |  |  |  |  |  |
| M2-M3-F3 | 2.632 | 2 | 1.316 | 0.498 | 0.611 | 0.022 | 0.996 | 0.127 |
| Error | 116.209 | 44 | 2.641 |  |  |  |  |  |
| F-C-P*L-M-R | 0.150 | 4 | 0.037 | 1.455 | 0.223 | 0.062 | 5.821 | 0.435 |
| Error | 2.261 | 88 | 0.026 |  |  |  |  |  |
| F-C-P*M2-M3-F3 | 2.682 | 4 | 0.670 | 3.429 | 0.012 | 0.135 | 13.716 | 0.837 |
| Error | 17.205 | 88 | 0.196 |  |  |  |  |  |
| L-M-R*M2-M3-F3 | 0.188 | 4 | 0.047 | 0.589 | 0.671 | 0.026 | 2.357 | 0.188 |
| Error | 7.007 | 88 | 0.080 |  |  |  |  |  |
| F-C-P*L-M-R*M2-M3-F3 | 0.145 | 8 | 0.018 | 0.839 | 0.570 | 0.037 | 6.709 | 0.383 |
| Error | 3.798 | 176 | 0.022 |  |  |  |  |  |

**Supplementary Table 3. ANOVA effects for syntactic violation N2 time-window**

| Effect | Repeated Measures Analysis of Variance of syntactic violation in the N2 time-window | | | | | | | |
| --- | --- | --- | --- | --- | --- | --- | --- | --- |
|  | SS | Degr. of (Freedom) | MS | F | p | Partial eta-squared | Non-centrality | Observed power (alpha=0.05) |
| Intercept | 5.196 | 1 | 5.196 | 0.599 | 0.447 | 0.026 | 0.599 | 0.115 |
| Error | 190.982 | 22 | 8.681 |  |  |  |  |  |
| F-C-P | 0.707 | 2 | 0.353 | 1.827 | 0.173 | 0.077 | 3.653 | 0.361 |
| Error | 8.513 | 44 | 0.193 |  |  |  |  |  |
| L-M-R | 0.470 | 2 | 0.235 | 0.339 | 0.714 | 0.015 | 0.679 | 0.101 |
| Error | 30.460 | 44 | 0.692 |  |  |  |  |  |
| M2-M3-F3 | 3.548 | 2 | 1.774 | 0.240 | 0.787 | 0.011 | 0.480 | 0.085 |
| Error | 324.973 | 44 | 7.386 |  |  |  |  |  |
| F-C-P*L-M-R | 1.253 | 4 | 0.313 | 0.916 | 0.458 | 0.040 | 3.664 | 0.280 |
| Error | 30.088 | 88 | 0.342 |  |  |  |  |  |
| F-C-P*M2-M3-F3 | 0.236 | 4 | 0.059 | 0.296 | 0.880 | 0.013 | 1.184 | 0.113 |
| Error | 17.517 | 88 | 0.199 |  |  |  |  |  |
| L-M-R*M2-M3-F3 | 0.840 | 4 | 0.210 | 0.547 | 0.702 | 0.024 | 2.187 | 0.177 |
| Error | 33.783 | 88 | 0.384 |  |  |  |  |  |
| F-C-P*L-M-R*M2-M3-F3 | 0.648 | 8 | 0.081 | 0.304 | 0.964 | 0.014 | 2.435 | 0.147 |
| Error | 46.850 | 176 | 0.266 |  |  |  |  |  |

**Supplementary Table 4. ANOVA effects for target P3**

| Effect | Repeated Measures Analysis of Variance of target P3 | | | | | | | |
| --- | --- | --- | --- | --- | --- | --- | --- | --- |
|  | SS | Degr. of (Freedom) | MS | F | p | Partial eta-squared | Non-centrality | Observed power (alpha=0,05) |
| Intercept | 317.362 | 1 | 317.362 | 22.469 | 0.000 | 0.505 | 22.469 | 0.995 |
| Error | 310.739 | 22 | 14.125 |  |  |  |  |  |
| F-C-P | 1037.596 | 2 | 518.798 | 103.702 | 0.000 | 0.825 | 207.404 | 1.000 |
| Error | 220.122 | 44 | 5.003 |  |  |  |  |  |
| L-M-R | 0.551 | 2 | 0.275 | 0.280 | 0.757 | 0.013 | 0.559 | 0.091 |
| Error | 43.330 | 44 | 0.985 |  |  |  |  |  |
| S-1-2 | 117.776 | 2 | 58.888 | 18.580 | 0.000 | 0.458 | 37.159 | 1.000 |
| Error | 139.457 | 44 | 3.169 |  |  |  |  |  |
| F-C-P*L-M-R | 17.486 | 4 | 4.372 | 14.561 | 0.000 | 0.398 | 58.244 | 1.000 |
| Error | 26.420 | 88 | 0.300 |  |  |  |  |  |
| F-C-P*S-1-2 | 5.167 | 4 | 1.292 | 2.901 | 0.026 | 0.116 | 11.603 | 0.762 |
| Error | 39.184 | 88 | 0.445 |  |  |  |  |  |
| L-M-R*S-1-2 | 1.350 | 4 | 0.337 | 3.973 | 0.005 | 0.153 | 15.891 | 0.892 |
| Error | 7.474 | 88 | 0.085 |  |  |  |  |  |
| F-C-P*L-M-R*S-1-2 | 0.623 | 8 | 0.078 | 2.575 | 0.011 | 0.105 | 20.598 | 0.911 |
| Error | 5.323 | 176 | 0.030 |  |  |  |  |  |

**Supplementary Table 5. ANOVA effects for distractor P3 time-window**

| Effect | Repeated Measures Analysis of Variance of distractor in the P3 time-window | | | | | | | |
| --- | --- | --- | --- | --- | --- | --- | --- | --- |
|  | SS | Degr. of (Freedom) | MS | F | p | Partial eta-squared | Non-centrality | Observed power (alpha=0,05) |
| Intercept | 11.146 | 1 | 11.146 | 4.372 | 0.048 | 0.166 | 4.372 | 0.516 |
| Error | 56.085 | 22 | 2.549 |  |  |  |  |  |
| F-C-P | 0.087 | 2 | 0.043 | 0.100 | 0.905 | 0.005 | 0.200 | 0.064 |
| Error | 19.098 | 44 | 0.434 |  |  |  |  |  |
| L-M-R | 0.037 | 2 | 0.018 | 0.124 | 0.884 | 0.006 | 0.247 | 0.068 |
| Error | 6.501 | 44 | 0.148 |  |  |  |  |  |
| M2-M3-F3 | 6.027 | 2 | 3.014 | 0.920 | 0.406 | 0.040 | 1.839 | 0.199 |
| Error | 144.168 | 44 | 3.277 |  |  |  |  |  |
| F-C-P*L-M-R | 0.114 | 4 | 0.028 | 1.090 | 0.366 | 0.047 | 4.362 | 0.330 |
| Error | 2.294 | 88 | 0.026 |  |  |  |  |  |
| F-C-P*M2-M3-F3 | 0.653 | 4 | 0.163 | 0.686 | 0.603 | 0.030 | 2.745 | 0.215 |
| Error | 20.932 | 88 | 0.238 |  |  |  |  |  |
| L-M-R*M2-M3-F3 | 0.348 | 4 | 0.087 | 1.023 | 0.400 | 0.044 | 4.093 | 0.311 |
| Error | 7.472 | 88 | 0.085 |  |  |  |  |  |
| F-C-P*L-M-R*M2-M3-F3 | 0.100 | 8 | 0.013 | 0.578 | 0.795 | 0.026 | 4.627 | 0.263 |
| Error | 3.805 | 176 | 0.022 |  |  |  |  |  |

**Supplementary Table 6. ANOVA effects for syntactic violation P3 time-window**

| Effect | Repeated Measures Analysis of Variance of syntactic violation in the P3 time-window | | | | | | | |
| --- | --- | --- | --- | --- | --- | --- | --- | --- |
|  | SS | Degr. of (Freedom) | MS | F | p | Partial eta-squared | Non-centrality | Observed power (alpha=0.05) |
| Intercept | 7.322 | 1 | 7.322 | 0.898 | 0.354 | 0.039 | 0.898 | 0.148 |
| Error | 179.385 | 22 | 8.154 |  |  |  |  |  |
| F-C-P | 0.106 | 2 | 0.053 | 0.198 | 0.821 | 0.009 | 0.396 | 0.079 |
| Error | 11.815 | 44 | 0.269 |  |  |  |  |  |
| L-M-R | 0.296 | 2 | 0.148 | 0.273 | 0.763 | 0.012 | 0.546 | 0.090 |
| Error | 23.840 | 44 | 0.542 |  |  |  |  |  |
| M2-M3-F3 | 35.624 | 2 | 17.812 | 3.774 | 0.031 | 0.146 | 7.547 | 0.659 |
| Error | 207.690 | 44 | 4.720 |  |  |  |  |  |
| F-C-P*L-M-R | 1.000 | 4 | 0.250 | 0.463 | 0.763 | 0.021 | 1.851 | 0.155 |
| Error | 47.522 | 88 | 0.540 |  |  |  |  |  |
| F-C-P*M2-M3-F3 | 0.501 | 4 | 0.125 | 0.788 | 0.536 | 0.035 | 3.151 | 0.243 |
| Error | 13.987 | 88 | 0.159 |  |  |  |  |  |
| L-M-R*M2-M3-F3 | 1.191 | 4 | 0.298 | 1.221 | 0.308 | 0.053 | 4.883 | 0.368 |
| Error | 21.459 | 88 | 0.244 |  |  |  |  |  |
| F-C-P*L-M-R*M2-M3-F3 | 3.520 | 8 | 0.440 | 1.870 | 0.067 | 0.078 | 14.962 | 0.777 |
| Error | 41.407 | 176 | 0.235 |  |  |  |  |  |

**Supplementary results: Testing the effects of the gender of the participants**

For testing the effects of the participant’s gender, all main analyses were re-run with the GENDER of the participant as a grouping variable. Regarding the behavioral results, there was no main effect or interaction with condition of participants’ GENDER on any behavioral measures (p>.58, all), except for the reaction time, where only main effect but no interaction occurred (p = .032). This was due to that female participants were generally faster than male ones. For the target and distractor ERPs in the N2 time window, no main effect or interaction was found for Gender (p > .161, at least). For the targets in the P3 time window, a main effect of GENDER was found (p = .018), and interaction with LATERALITY (p = .007) and ANTERIOR-POSTERIOR (p < .001), but there was no interaction with CONDITION (p > .071). For the distractors, no main effect or interaction was found with GENDER (p > .129).
